# Case report: A pediatric case of Bickerstaff brainstem encephalitis after COVID-19 vaccination and Mycoplasma pneumoniae infection: Looking for the culprit

**DOI:** 10.3389/fimmu.2022.987968

**Published:** 2022-08-12

**Authors:** Gabriele Monte, Stefano Pro, Fabiana Ursitti, Michela Ada Noris Ferilli, Romina Moavero, Laura Papetti, Giorgia Sforza, Giorgia Bracaglia, Federico Vigevano, Paolo Palma, Massimiliano Valeriani

**Affiliations:** ^1^ Neurology Unit, Department of Neuroscience, Bambino Gesù Children’s Hospital, Istituto di Ricovero e Cura a Carattere Scientifico (IRCCS), Rome, Italy; ^2^ Child Neurology and Psychiatry Unit, Department of System Medicine, Tor Vergata University of Rome, Rome, Italy; ^3^ Department of Diagnostics and Laboratory Medicine, Medical Laboratory Unit, Unit of Allergy and Autoimmunity, Bambino Gesù Children’s Hospital, Istituto di Ricovero e Cura a Carattere Scientifico (IRCCS), Rome, Italy; ^4^ Academic Department of Pediatrics (DPUO), Research Unit of Clinical Immunology and Vaccinology, Bambino Gesù Children’s Hospital, Istituto di Ricovero e Cura a Carattere Scientifico (IRCCS), Rome, Italy; ^5^ Chair of Pediatrics, Department of Systems Medicine, Tor Vergata University of Rome, Rome, Italy; ^6^ Center for Sensory-Motor Interaction, Aalborg University, Denmark Neurology Unit, Aalborg, Denmark

**Keywords:** COVID-19 vaccination, immune-mediated diseases, Bickerstaff brainstem encephalitis, anti-GQ1b antibody, Mycoplasma pneumoniae

## Abstract

Bickerstaff brainstem encephalitis (BBE) is a rare, immune-mediated disease characterized by the acute onset of external ophthalmoplegia, ataxia, and consciousness disturbance. It has a complex multifactorial etiology, and a preceding infectious illness is seen in the majority of cases. Immune-mediated neurological syndromes following COVID-19 vaccination have been increasingly described. Here we report the case of a child developing BBE 2 weeks after COVID-19 vaccination. Despite nerve conduction studies and CSF analysis showing normal results, BBE was diagnosed on clinical ground and immunotherapy was started early with a complete recovery. Later, diagnosis was confirmed by positive anti-GQ1b IgG in serum. Even if there was a close temporal relationship between disease onset and COVID-19 vaccination, our patient also had evidence of a recent Mycoplasma pneumoniae infection that is associated with BBE. Indeed, the similarity between bacterial glycolipids and human myelin glycolipids, including gangliosides, could lead to an aberrantly immune activation against self-antigens (i.e., molecular mimicry). We considered the recent Mycoplasma pneumoniae infection a more plausible explanation of the disease onset. Our case report suggests that suspect cases of side effects related to COVID-19 vaccines need a careful evaluation in order to rule out well-known associated factors before claiming for a causal relationship.

## Introduction

Bickerstaff brainstem encephalitis (BBE) is a rare, immune-mediated disease characterized by the acute onset of external ophthalmoplegia, ataxia, and consciousness disturbance. In addition to this characteristic triad, areflexia, extremity weakness, sensory alterations, and bulbar and facial palsy are frequently reported (1, 2). Serum anti-GQ1b IgG antibodies (Abs) are detected at different frequencies in patients with BBE (3) and represent a feature common to Guillain–Barrè syndrome (GBS) and Miller Fisher syndrome (MFS) (4). Indeed, it is generally thought that BBE is not a distinct neurological entity but lies at one end of a spectrum of diseases known as the anti-GQ1b syndromes. A preceding infectious illness is seen in the majority of BBE cases (5, 6) and is considered a potential trigger of the autoimmune response. Epitopes present on Campylobacter jejuni (7) and Mycoplasma pneumoniae (8) share a marked similarity with human myelin glycolipids, including gangliosides. Therefore, Abs elicited by the aforementioned infective agents could cross-react with structurally similar gangliosides, leading to off-target immune-mediated tissue damage in susceptible individuals. Another possible trigger of autoimmunity is vaccination, which is thought to act through a strong induction of proinflammatory cytokines and T-cell response (9). The association between vaccination and immune-mediated neurological syndromes made a comeback during the Coronavirus Disease 19 (COVID-19) pandemic. Neurological complications following vaccination have been increasingly described (10). Here we report the case of a child developing BBE a few days after the second dose of COVID-19 vaccination (Pfizer-BioNTech).

## Case description

A 15-year-old man was admitted to our Emergency Department for acute onset of asthenia, limb paresthesia, and gait unsteadiness followed in the next day by diplopia, dysarthria, and consciousness disturbance. Two weeks before the disease onset, he received the second dose of SARS-CoV2 vaccination. Ten days before admission, the patient had fever, coughing, and vomiting that resolved spontaneously within 3 days, and the nasopharyngeal swab test for SARS-CoV-2 was negative.

**Figure 1:**
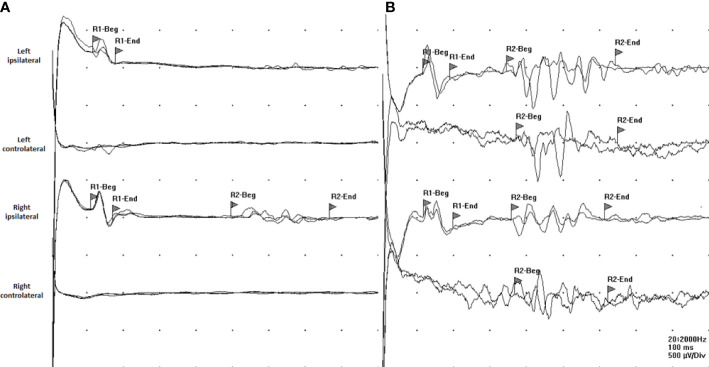
Recording from both orbicularis oculi muscles, stimulating the supraorbital nerve on each side. **(A)** Stimulating the left side results in a normal R1 response while ipsilateral and contralateral R2 components are absent. Stimulating the right side results in a normal R1 component, while the ipsilateral R2 response is delayed and the contralateral R2 component is absent. This feature is related to a bilateral alteration of the multisynaptic pathway between the nucleus of the spinal tract of V cranial nerve in the ipsilateral pons and medulla and interneurons forming connections to the ipsilateral and contralateral facial nuclei. **(B)** The blink reflex is normal.

Neurological examination showed a stuporous state, dysarthria, vertical ophthalmoplegia, VI and VII left cranial nerve paresis with lateral-gaze diplopia, and limb hypotonia with normal deep tendon reflexes and no meningeal signs. The patient had no fever, and he resulted negative for SARS-CoV2 infection. Magnetic resonance imaging (MRI) of the brain and spinal cord with contrast was normal. Lumbar puncture was performed, and cerebrospinal fluid (CSF) analysis showed normal results. CSF cultures and polymerase chain reaction (PCR) showed no evidence of infection. Oligoclonal bands resulted negative. The electroencephalogram (EEG) showed an alpha activity with poor regional differentiation consistent with an “alpha coma pattern”. Nerve conduction studies, F-response, and H-reflex resulted normal while blink reflex showed an abnormal pattern suggesting brainstem dysfunction (**Figure 1A**). Routine laboratory examination was normal, and toxicological screening was negative. Anti-Mycoplasma pneumoniae IgM and low titer IgG (1.7 U/ml, normal value <0.9 U/ml) were detected in serum, while PCR for Mycoplasma pneumoniae resulted negative in throat specimen. Anti-SARS-CoV2 serological response was evaluated, and both anti-spike and anti-nucleocapsid Abs resulted positive, suggesting a previous unknown infection. Chest X-ray was normal. The tests performed on blood, cerebrospinal fluid, nasopharyngeal and urine samples are reported in **Table 1**.

**Table 1:** Test performed on blood, cerebrospinal fluid, nasopharyngeal, and urine samples.

Sample	Test
Blood	Cell count, routine chemical exam, culture IgM and IgG: CMV, EBV, HSV, HHV-6, MV, Mumps virus, Rubella virus, SARS-CoV2, VZV, Mycoplasma pneumoniae, Chlamydia pneumoniae AE panel: Anti-NMDAr Ab, Anti-AMPAr Ab, Anti-CASPR2 Ab, Anti-LGI1 Ab, Anti-GABAr Ab, Anti-DPPX Ab, onconeural Abs
Cerebrospinal fluid	Cell count, protein, glucose, lactate, oligoclonal bands, cytological examination Culture, PCR for bacterial (Streptococcus pneumoniae and agalactiae, Neisseria meningitidis, Listeria monocytogenes, Haemophilus influenzae, Escherichia coli) and virus infection (CMV, EBV, Enterovirus, HSV, Rubella virus, VZV) AE panel: Anti-NMDAr Ab, Anti-AMPAr Ab, Anti-CASPR2 Ab, Anti-LGI1 Ab, Anti-GABAr Ab, Anti-DPPX Ab, onconeural Abs
Nasal and pharyngeal swab	PCR for virus (SARS-CoV2) and bacterial infection (Mycoplasma pneumoniae, Chlamydia pneumoniae)
Urine	Cell count, protein, glucose, blood cell count, leukocyte esterase, nitrites, culture Toxicological screening

Ab, antibody; AE, autoimmune encephalitis; AMPAr, α-amino-3-hydroxy-5-methyl-4-isoxazolepropionic acid receptor; CASPR2, contactin-associated protein-2; CMV, Cytomegalovirus; DPPX, dipeptidyl-peptidase-like protein 6; EBV, Epstein–Barr virus; GABA, gamma aminobutyric acid receptor; HHV, human herpesvirus; HSV, herpes simplex virus; Ig, immunoglobulin; LGI1, leucine-rich glioma inactivated 1; MV, measles virus; NMDAr, N-methyl-d-aspartate receptor; PCR, polymerase chain reaction; VZV, varicella-zoster virus.

**Figure 2:**
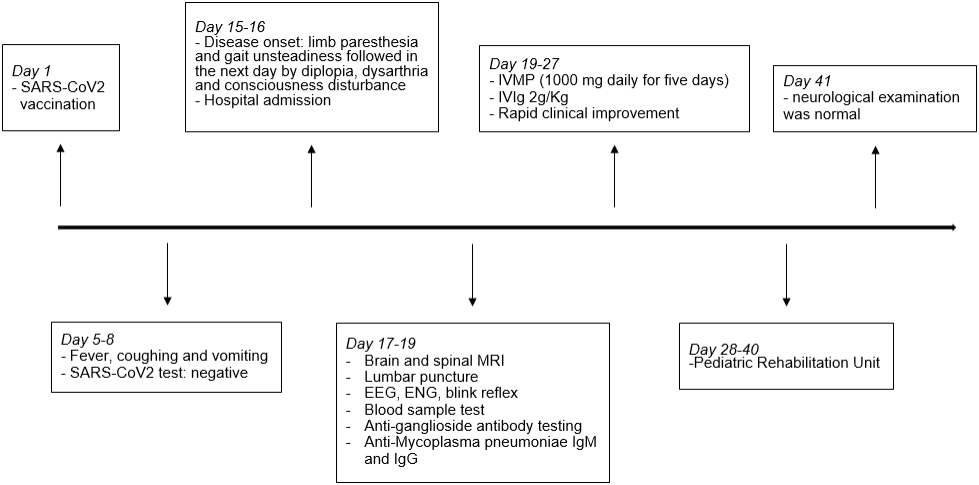
Timeline with selected events. EEG, electroencephalogram; ENG, electroneurography; IVIg, intravenous immunoglobulin; IVMP, intravenous methylprednisolone; MRI, magnetic resonance imaging.

Overall, these findings were consistent with BBE and the patient was treated with intravenous methylprednisolone 1,000 mg daily for 5 days plus intravenous immunoglobulins (IVIg) 2 g/kg with a rapid improvement of consciousness and gradual resolution of the ophthalmoplegia and facial palsy. Due to the persistence of gait ataxia, the patient was still unable to walk without assistance. MRI of the brain and spinal cord with contrast was repeated and showed a normal result, as well as EEG activity. Anti-ganglioside Ab testing revealed positive anti-GQ1b IgG in serum, confirming the diagnosis of BBE. The patient was transferred to the Pediatric Rehabilitation Unit. After 2 weeks, he was able to walk without assistance and the neurological examination was normal (**Figure 2**). Six weeks after disease onset, the blink reflex resulted normal (**Figure 1B**).

## Discussion

Immune-mediated diseases have a complex multifactorial etiology, and many factors can contribute to their onset. Infectious agents are among the most important environmental triggers of autoimmunity, through various mechanisms such as molecular mimicry, bystander activation, and polyclonal activation (11).

Two weeks before the disease onset, our patient received the second dose of SARS-CoV2 vaccination (Pfizer-BioNTech), a temporal relationship that may suggest a possible causal link. Neurological immune-related adverse events, especially GBS and its variants, have been reported after COVID-19 vaccination (10). GBS has been associated with some vaccination programs (e.g., for influenza), but the risk was shown to be very small when weighed against the benefits of immunization (12). A recent paper analyzed National Health Service (NHS) data on GBS cases and COVID-19 vaccination in England and found no increased risk in people receiving Comirnaty (Pfizer-BioNTech) (13). Furthermore, in the past, most associations between vaccines and nervous system autoimmune syndromes that have been reported as severe adverse events following immunization were no longer evidenced when well-conducted epidemiological studies were carried out (14). Notably, to improve the reliability of COVID-19 vaccination post-marketing surveillance and the causal relationship assessment of post-immunization adverse events, it is of outmost importance to exclude commonly associated causes and triggers of immune-mediated neurological syndromes (15). Mycoplasma pneumoniae is a major cause of respiratory tract infections in children and has been associated with BBE (16, 17). The similarity between bacterial glycolipids and host myelin glycolipids, including gangliosides, could lead to an aberrantly immune activation against self-antigens (i.e., molecular mimicry) (8, 11). Our patient presented 10 days after a respiratory illness and had laboratory evidence of recent Mycoplasma pneumoniae infection, both supporting its role as a trigger of BBE. In conclusion, in the case reported here, even if there was a close temporal relationship between BBE onset and SARS-CoV2 vaccination, the aforementioned findings—providing a more plausible explanation—make a causal correlation unlikely.

Importantly, in our case nerve conduction studies and CSF analysis showed normal results, as already reported in the early stages of the disease. These findings should not delay treatment if the diagnosis is highly suspected on clinical grounds (18), given that the early use of immunotherapy is associated with a better outcome (2).

In conclusion, this case highlights the importance of prompt recognition and diagnosis of such rare disease to ensure effective management and treatment. Even if this is a single case report, we suggest that suspect cases of COVID-19 vaccine-related side effects should firstly be carefully analyzed to rule out well-known associated factors before claiming for a causal relationship. Overall, vaccination should still be advocated considering the markedly increased risk of complications—including postinfectious autoimmune disorders—after SARS-CoV2 infection.

## Data availability statement

The raw data supporting the conclusions of this article will be made available by the authors, without undue reservation.

## Ethics statement

Ethical review and approval was not required for the study on human participants in accordance with the local legislation and institutional requirements. Written informed consent to participate in this study was provided by the participants’ legal guardian/next of kin. Written informed consent was obtained from the minor(s)’ legal guardian/next of kin for the publication of any potentially identifiable images or data included in this article.

## Author contributions

GM, FU, MF, RM, LP, GS and GB acquired the clinical data. SP performed neurophysiological studies. GM and MV drafted the manuscript. GM, MV, PP and FV critically revised the manuscript. All authors approved the final version of the manuscript.

## Conflict of interest

The authors declare that the research was conducted in the absence of any commercial or financial relationships that could be construed as a potential conflict of interest.

## Publisher’s note

All claims expressed in this article are solely those of the authors and do not necessarily represent those of their affiliated organizations, or those of the publisher, the editors and the reviewers. Any product that may be evaluated in this article, or claim that may be made by its manufacturer, is not guaranteed or endorsed by the publisher.
